# Whole Exome Sequencing of an X-linked Thrombocytopenia Patient with Normal Sized Platelets

**Published:** 2019

**Authors:** Majid Fathi, Hojat Shahraki, Edris Sharif Rahmani, Hamzeh Rahimi, Pouria Omidi, Saeedeh Darvishi, Mohammad Foad Abazari, Arshad Hosseini

**Affiliations:** 1. Department of Medical Biotechnology, Faculty of Allied Medicine, Iran University of Medical Sciences, Tehran, Iran; 2. Department of Laboratory Sciences, Faculty of Allied Medicine, Zahedan University of Medical Sciences, Zahedan, Iran; 3. Clinical Immunology Research Center, Zahedan University of Medical Sciences, Zahedan, Iran; 4. Department of Molecular Medicine, Biotechnology Research Center, Pasteur Institute of Iran, Tehran, Iran; 5. Department of Clinical Pathology, Faculty of Veterinary Medicine, University of Tehran, Tehran, Iran; 6. Department of Genetics, Tehran Medical Sciences Branch, Islamic Azad University, Tehran, Iran

**Keywords:** Blood platelets, Thrombocytopenia, Whole exome sequencing, Wiskott-Aldrich syndrome

## Abstract

Wiskott-Aldrich Syndrome (*WAS*) is a rare X-linked recessive Primary Immunodeficiency (PID) caused by mutations in *WAS* gene which encodes a protein known as WASp. WASp plays important roles in cytoskeletal functions that compromise multiple aspects of normal cellular activity including proliferation, phagocytosis, immune synapse formation, adhesion and directed migration. WASp defect particularly causes platelets abnormality which is presented in forms of decrease of Mean Platelet Volume (MPV) and thrombocytopenia in most *WAS* conditions; nevertheless, some studies reported *WAS* patients with a normal or large size of platelets in recent years. This phenomenon is unique and the exact mechanism of thrombocytopenia with a normal or large size of platelets is still unknown. In this study, Next Generation Sequencing (NGS) was utilized to discover the causing mutation in *WAS* gene; furthermore, an attempt was made to evaluate the possibility of other mutations or genes especially WASp interacting proteins and inherited platelet disorder genes in patient clinical symptoms for the purpose of understanding the origin of such unique symptom and to perform further analysis if it is required. Therefore, clinical manifestations and immunologic functions of the patient were checked and Whole Exome Sequencing (WES) was performed to analyze all exonic variations which can be associated with patient phenotypes. Finally, a novel de novo mutation in *WAS* gene which truncates WASp to half of its normal size was determined as the only cause of clinical manifestation.

## Introduction

*Wiskott-Aldrich Syndrome (WAS)* gene is located on a petite arm of chromosome X (X: 48,683,752-48,691,426) and consists of 12 exons (1823 *bp*) encoding a 502 amino acid cytosolic protein named WASp. WASp is constitutively expressed in all hematopoietic stem-cell-derived lineages, except in mature red blood cells 1–3. Neural WASp (N-WASp) and WASp family verprolin homologous protein 1 (WAVE1), WAVE2 and WAVE3 are other actin-cytoskeleton reorganizers that are more widely expressed 4.

The incidence of *WAS* is approximately 1 in 250,000 male births and this condition is rarer in females 5. At least, 400 different disease-causing mutations have been identified in *WAS* gene scattered throughout all 12 exons sequences, although as many as nine mutational hot spots have been identified that account for about one-third of the total number of reported mutations 6. These mutations result in loss or gain of WASp function and type of mutation strongly influences the clinical severity of the disease. Generally, they result in three types of phenotypes, the most severe form is the classic *WAS* triad of thrombocytopenia, small platelets/recurrent infections and eczema. The milder form is X-Linked Thrombocytopenia (XLT) characterized by persistent thrombocytopenia with minimal or no sign of eczema and immunodeficiency. The third scarce one is X-Linked Neutropenia (XLN) without any of the clinical findings of *WAS/XLT* which is due to gain of function mutations of *WAS* gene that result in constitutively activated WASp. Only four missense mutations have been described for XLN as L270P, S272P, I294T and I290T, all located in exon 9 7–9. A system of scoring has been established in 1995 to describe the severity of the disease based on the symptoms (Table 1) 10.

**Table 1.** Scoring system of WAS: −/(+) absent or mild, (+) mild, transient eczema or mild transient infections not resulting in sequelae, + persistent, but therapy responsive eczema and recurrent infections requiring antibiotics and often intravenous immunoglobulin prophylaxis, ++ eczema that is difficult to control and severe threatening infections

	IXLT	XLT	Classic	WAS	XLN
<1	1	2	3	4	5	0
Thrombocytopenia	−/+	+	+	+	+	+	−
Small platelets	−	+	+	+	+	+	−
Eczema	−	−	(+)	+	++	(+)/+/++	−
Immunodeficiency	−	−/(+)	(+)	+	+	(+)/+	−
Infections	−	−	(+)	+	+/++	(+)/+/++	−
Autoimmunity and/or malignancy	−	−	−	−	−	+	−
Congenital neutropenia	−	−	−	−	−	−	+

1 Because patients with XLT may develop autoimmune disorders or lymphoma, albeit at a lower rate than those with Classic WAS, progression from a score of 1 or 2 to a 5 is possible for XLT.

In spite of our current extensive progress in the molecular basis of *WAS/XLT* circumstances, a prominent heterogeneity has still remained in its clinical presentations and laboratory findings. In *WAS* classes with a score of ≥1, microthrombocytopenia that is thrombocytopenia with small platelet size, is a key criterion in diagnosis but recent anecdotal cases with mutations in *WAS* gene displayed normal or large size of platelets leading to misinterpretation and false diagnosis in this patient 11,12. Normal platelet size (MPV 7-11 *fL*) with thrombocytopenia is the characteristic of some platelet disorders like cyclic thrombocytopenia and congenital amegakaryocytic thrombocytopenia. Giant platelets (>11 *fL*) with thrombocytopenia is presented with diseases like Gray platelet syndrome or GATA1-related cytopenia 13. Abnormality of platelets size in *WAS/XLT* patients is not limited to recent studies and even two studies reported cases with a large and normal size of platelets in the nineties 14,15. A unique form of this phenotype raised suspicion for the contingency of an associated novel genetic variation not necessarily in *WAS* gene but multiple alleles from other genes that could collaborate with *WAS* mutations.

In the present study, an Iranian boy suspected of XLT with normal MPV was found, so the genomic alteration mechanism was evaluated by investigating genes variations with next generation sequencing (Exome sequencing). Our results showed no other specific mutation to attribute to our patient phenotype. Therefore, it seems sequencing of coding regions of the genome cannot explain the observation of this unusual occurrence but future works with complementary experiments like examining patient transcripts or epigenetic analysis may resolve this issue and facilitate recognition of these patients for prompt treatment.

## Materials and Methods

### Characterization of the patient

Our protocol was performed with the approval of Iran University of Medical Sciences (IUMS) and written informed consent for genetic testing was obtained from the patientņs parents in accordance with the guidelines of IUMS research ethics committee. The patient was a seven year old boy with clinical manifestations of moderate thrombocytopenia, periodic almost monthly infections (conjunctivitis, otitis, gingivitis) and diarrhea which lasted approximately one week; he did not show eczema or bleeding diathesis. He was from consanguineous parents (maternal cousins) and he had a 17 year old sister with no signs of immunodeficiency or platelet disorder. Also, pedigree analysis demonstrated no family history of immune system disorder or severe infections among his family members.

During the last two years, Complete Blood Count (CBC) had been measured six times and all of them displayed normal MPV (mean 8.6 *fL*). At the time of referral, the white blood cell count was 8.02 (×10^3^/*µl*) with 27.4% lymphocytes and 58.3% neutrophils; hemoglobin 11.8 (*gr/L*), platelets (Plt) 31 (×10^3^/*µl*) and MPV of 8.3 *fL*. Serum levels of immunoglobulin for IgG, IgA, IgM and IgE were 2.123 (*g/L*), 0.261 (*g/L*), 0.058 (*g/L*) and 27.9 *IU/ml*), respectively. Flow cytometry of peripheral blood leukocytes showed a severe decrease of CD4+T lymphocytes [0.983 (10^9^/*L*)] and a milder decrease of CD3+T lymphocytes [0.684 (10^9^/*L*)] and CD8+T lymphocytes [0.675 (10^9^/*L*)]. According to his presentations and laboratory results, he was a candidate for XLT and received treatments as Intravenous Immune Globulin (IVIG) therapy and prophylaxis antibiotics like cotrimoxazole and allogeneic Hematopoietic Stem Cell Transplantation (HSCT) from his sister had been advised for his permanent cure but his parents refused further treatment. Sanger sequencing was also performed for some of the patient relatives and eight healthy controls with no history of immune or platelet disorders.

### DNA extraction and exome sequencing

DNAs of patient and his parents were isolated from EDTA anti-coagulated blood by QIAamp DNA Minikit (Qiagen) and exome library preparation was accomplished by SureSelect^xt^ Target Enrichment System (Agilent Technologies, Santa Carla, CA, USA), then captured fragments were massive parallel paired-end sequenced on an Illumina Hiseq 4000 platform on average coverage of 150×. The process of Whole Genome Sequencing (WES) was performed by Macrogen Company (Geumcheon-gu, Seoul, South Korea).

### WES analysis

Our WES analysis workflow involved quality control, alignment, variant detection, prioritization, and an-notation. At first, quality of sequenced reads were checked and trimmed with Fastx_toolkit (version 0.7, http://hannonlab.cshl.edu ), subsequently remaining reads were mapped against the latest human reference genome NCBI build GRch38 (University of California Santa Cruz human genome assembly) using BWA (Version 0.7.12, http://bio-bwa.sourceforge.net ) and re-moving duplicates was completed by Picard tools (Version 2.13.2, http://picard.sourceforge.net/ ) and after re-dundancy filtering, variants were called with SAM tools (Version 1.5, http://samtools.sourceforge.net ).

Variants filtering and annotation was performed with KGGSeq (Version 1.0+, http://grass.cgs.hku.hk ). Filtering was based on parameters like keeping variants with coverage ≥15, minor allele coverage ≥10 and variant call quality ≥20 and filtering of others. Also, common non-disease known genetic variants from genome variation projects data (1000 genomes project, Exome Aggregation Consortium (ExAC r0.3.1), http://exac.broadinstitute.org ), DiscovEHR ( http://www.discove-hrshare.com ) and Genome Aggregation Database ( http://gnomad.broadinstitute.org ) and variants with Minor Allele Frequency (MAF) ≥0.01 were excluded. In the final stage of analysis, variants were annotated utilizing different databases like dbSNP ( https://www.ncbi.nlm.nih.gov/projects/SNP ), dbscSNV ( https://sites.google.com/site/jpopgen/dbNSF ) OMIM ( https://www.omim.org ), Cosmic ( http://cancer.sanger.ac.uk/cosmic ) and PubMed ( https://www.ncbi.nlm.nih.gov/pubmed ) and also with some computational predicting algorithms such as CADD, SIFT, Polyphen, FATHMIM and Mutation taster, the degree of deleteriousness of each allele was calculated.

All types of mutations of WASp interacting proteins (about 10 proteins which are associated directly or indirectly) and inherited platelet disorder genes (76 genes) that are potentially prone genes for our patient phenotype were particularly scrutinized to find the minimum possible condition required for malady 16,17.

### PCR amplification and Sanger sequencing

Candidate variant of WES analysis needs to be independently confirmed by comparison of Sanger sequencing results of the patient, his kindred and control individuals. For proceeding further, primer sequences of the PCR were designed by Primer3 (Version 0.4.0, http://bioinfo.ut.ee/primer3-0.4.0 ) and region of candidate variant including mutation and its flanking regions were amplified by standard PCR.

The PCR conditions were as follows; initial denaturation at 95*°C* for 5 *min*, then 35 cycles of denaturation at 95*°C* for 30 *s*, annealing at 62*°C* for 30 *s*, elongation at 72*°C* for 30 *s* and extension at 72*°C* for 5 *min*.

## Results

After WES, more than 110 million 101-*bp* reads were generated for each sample. Quality control results were appropriate and reliable for next analysis as average GC content was 49.08 and bases with a Phred score >30 were 97.98% and ambiguous bases were near to 0%. For alignment, 99.98% of the reads were mapped to the reference genome. In the following, a total of 103,903 variants were called then common nonpathogenic variants were filtered out by evaluation of functional impact and using databases described above. Finally, 76 potentially damaging variants were identified.

Most of the remaining variants were missense and totally they could be responsible for six kinds of phenotypes including the neurological function and development defects, renal and urinary system defects, vision and eye defects, deafness phenotype and hematopoietic system defects. According to the patient manifestations and also studying cellular and molecular pathways of each variant, only hematopoietic system variants could be associated with the current features of the patient and eventually a frame shift mutation (c.A685del-G, p.G229fs*260) in *WAS* gene with chromosomal coordination of X:48686905 was considered as the pathogenic variant. This mutation results in a stop codon in amino acid 260 that truncates the encoded protein to half of its size. Precise investigation of WASp-interacting proteins and inherited platelet disorder genes revealed no significant mutation that could support the presence of other diseases besides *WAS*.

Sanger sequencing also confirmed this deletion. *WAS* mutation was checked in both maternal and paternal grandparents and eight control individuals. All of them were not mutated and only the patient, his mother and his sister exhibited defective allele which defined the mother as the origin of the mutation.

WASp expression in lymphocytes was evaluated by flow cytometry in the next follow up appointment that was negative in the patient as compared to a healthy control.

## Discussion

Wiskott-Aldrich syndrome was first described by Alfred Wiskott in 1933 and then Robert Aldrich showed an X-linked recessive inheritance pattern of the disease with segregation analysis in 1954 18. Four decades later, disease causing gene was isolated by positional cloning strategy in 1994 19 and since then different studies were conducted to understand the effect of location and type of each *WAS* variant on the severity of the disease. A cohort study of 270 unrelated *WAS/XLT* families from single centers in the United States, Italy, and Japan represented a consistent phenotype-genotype correlation as patients with mutations that resulted in expression of normal-sized mutated protein, often in reduced quantity, manifest, with few exceptions, the XLT phenotype, whereas those patients whose lymphocytes could not express WASp or express only truncated WASp were more likely to have the classic *WAS* phenotype 20.

WASp consists of five functional domains of Ena-VASP homology domain also known as WASp homology domain (WH1) at the N-terminal followed by a basic region (BR), a GTPase Binding Domain (GBD), a proline rich sequence and a Verproline homology, cofilin homology and acidic region (VCA) domain at the C-terminal 2. WAS pinteracting protein (WIP) interacts with the N-terminal WH1 domain which regulates its stability, by preventing posttranslational degradation, and C-terminal domain activates actin poly-merization via Actin Related Protein (Arp2/3) complex 2,21. In other conditions, the VCA domain interacts with the GBD region and is not available to the Arp2/3 complex. Upon cell activation, binding of Cdc42-GTP to the GBD results in the release of the VCA domain, thus mediating actin polymerization 6.

Thrombocytopenia and small platelets volume are consistent features of *WAS* and XLT, irrespective of the severity of the disease, but its pathogenesis still remains controversial 22. WASp is required for various platelet cellular processes like regulation of podosome formation in megakaryocytes and in human this is associated with a defect in platelet count, size and morphology 23. In the absence of WASp, actin polymerization became defective and Megakaryocytes (MKs) could release platelets prematurely which leads to their accumulation in bone marrow 24. Initial studies have shown that platelets from WAS patients are irregular in shape and structure, lack pseudopodia, spread poorly and their F-actin content is decreased. They also have fewer alpha granules in their cytoplasm and aggregate poorly. Interestingly, later studies reported normal platelet activation and aggregation, procoagulant activity, alpha-granule secretion, filopodia extension, lamellae spreading, F-actin increase, and Arp2/3 complex activation which is supposed to be related to splenectomy 25.

However, increase or normal size of platelets was observed in infrequent *WAS/XLT* cases with no splenectomy or other previous known events which could affect platelet size. This phenomenon makes disease diagnosis complicated like misdiagnosis of *WAS/XLT* with other platelet disorders mostly Immune Thrombocytopenia (ITP) and due to uncertainty of MPV in practice, which may hinder the diagnosis of *WAS*, assessment of Immature Platelet Fraction (IPF) instead of MPV is advised to prevent inappropriate treatment and delay life-saving therapy 26,27.

The pathogenesis of normal or large size platelets in WAS patients is thoroughly unknown and has been mentioned by a few studies but malignant transformation of *WAS* or concomitant disorders are assumed to be more liable for the phenotype, like presence of autoimmune disease or Immune Thrombocytopenia Purpura (ITP) usually due to infections, drugs and vaccination in comparison to other rare hereditary causes such as Bernard-Soulier syndrome, DiGeorge/Velo-cardiofacial syndrome or Platelet-type von Willebrand disease based on clinical and laboratory findings 11.

A previous study utilized next generation sequencing for a child presenting with macrothrombocytopenia to find genetic variation. In addition to *WAS* gene, a total of 70 additional genes known to harbor variants implicated in Inherited Platelet Disorders (IPDs) were further target sequenced and at last there was no identification of other defects, but there is the possibility of coexistence of a still unidentified genetic variant which may be recognized by multigene high-throughput sequencing 28.

Assessment of our under study patient manifestations exhibited none of the hypothesized diseases which may contribute to platelet normal size; so, the patient was genetically analyzed by means of WES as one of NGS methods looking forward to finding pathogenic variations which might be the reason of abnormal size of platelets in *WAS/XLT* patients and subsequently their likely roles were investigated by functional analysis. To date, over 300 deletions, insertion and splice site mutation in the *WASP* gene have been reported to cover all exons 29–31. Over 400 patients with WASP gene mutation have been reported to date. The mutations, WASP expressions and the phenotypes reported are accessible on the Internet as WASP base 32. Our results only manifest *WAS* mutation, and deletion of one nucleotide (Guanine) in exon 7 of *WAS* gene (Frameshift mutation) in amino acid 260 stop codon. *WAS* 685delG mutation causes partial deletion of WASp after the GBD domain although EVH1 domain remains intact for binding to WIP. Previous studies indicated that a mutant WASp with normal interaction with WIP is relatively stable which protects the truncated protein from being degraded 33 but due to the lack of VCA area, truncated WASp cannot combine to the Arp2/3 protein complex. Therefore, WASp expression was absent in our patient as measured by flow cytometry. Sanger sequencing of patient and his kindred unraveled the origin of mutation as a heterozygous *de novo* deletion in his mother. His sister also showed the same allele as her mother. This novel mutation was not reported in any of other SNP databases but its stop codon in amino acid 260 was previously reported 34.

## Conclusion

To our knowledge, all coding regions of a patient who is discordant with *WAS/XLT* phenotype were analyzed for his manifestation of normal sized platelets for the first time and the result was only *WAS* mutation like other typical patients. This finding can encourage presumption of co-occurrence of other phenotypes as the causing factor; however, other genes expression and epigenetic alteration of *WAS* gene are two important incidences that have not been evaluated in this study which may improve our understanding of the spectrum of clinical and phenotypic heterogeneity 35.
